# Imaging Mass Spectrometry Reveals Acyl-Chain- and Region-Specific Sphingolipid Metabolism in the Kidneys of Sphingomyelin Synthase 2-Deficient Mice

**DOI:** 10.1371/journal.pone.0152191

**Published:** 2016-03-24

**Authors:** Masayuki Sugimoto, Masato Wakabayashi, Yoichi Shimizu, Takeshi Yoshioka, Kenichi Higashino, Yoshito Numata, Tomohiko Okuda, Songji Zhao, Shota Sakai, Yasuyuki Igarashi, Yuji Kuge

**Affiliations:** 1 Department of Integrated Molecular Imaging, Graduate School of Medicine, Hokkaido University, Sapporo, Japan; 2 Shionogi Innovation Center for Drug Discovery, Discovery Research Laboratory for Innovative Frontier Medicines, Shionogi & Co., Ltd., Sapporo, Japan; 3 Central Institute of Isotope Science, Hokkaido University, Sapporo, Japan; 4 Laboratory of Bioanalysis and Molecular Imaging, Faculty of Pharmaceutical Science, Hokkaido University, Sapporo, Japan; 5 Drug Discovery Technologies, Discovery Research Laboratory for Core Therapeutic Areas, Shionogi & Co., Ltd., Toyonaka, Japan; 6 Department of Tracer Kinetics & Bioanalysis, Graduate School of Medicine, Hokkaido University, Sapporo, Japan; 7 Department of Biomembrane and Biofunctional Chemistry, Faculty of Advanced Life Science, Hokkaido University, Sapporo, Japan; Stony Brook University, UNITED STATES

## Abstract

Obesity was reported to cause kidney injury by excessive accumulation of sphingolipids such as sphingomyelin and ceramide. Sphingomyelin synthase 2 (SMS2) is an important enzyme for hepatic sphingolipid homeostasis and its dysfunction is considered to result in fatty liver disease. The expression of SMS2 is also high in the kidneys. However, the contribution of SMS2 on renal sphingolipid metabolism remains unclear. Imaging mass spectrometry is a powerful tool to visualize the distribution and provide quantitative data on lipids in tissue sections. Thus, in this study, we analyzed the effects of SMS2 deficiency on the distribution and concentration of sphingomyelins in the liver and kidneys of mice fed with a normal-diet or a high-fat-diet using imaging mass spectrometry and liquid chromatography/electrospray ionization-tandem mass spectrometry. Our study revealed that high-fat-diet increased C18–C22 sphingomyelins, but decreased C24-sphingomyelins, in the liver and kidneys of wild-type mice. By contrast, SMS2 deficiency decreased C18–C24 sphingomyelins in the liver. Although a similar trend was observed in the whole-kidneys, the effects were minor. Interestingly, imaging mass spectrometry revealed that sphingomyelin localization was specific to each acyl-chain length in the kidneys. Further, SMS2 deficiency mainly decreased C22-sphingomyelin in the renal medulla and C24-sphingomyelins in the renal cortex. Thus, imaging mass spectrometry can provide visual assessment of the contribution of SMS2 on acyl-chain- and region-specific sphingomyelin metabolism in the kidneys.

## Introduction

Imaging mass spectrometry (IMS) using matrix-assisted laser desorption/ionization-mass spectrometry (MALDI-MS) is a powerful tool for visualizing the distribution of various molecules in tissue sections [1–5]. The major advantage of IMS is its ability to simultaneously detect a number of molecules without the use of imaging probes. Lipids are essential for various biological functions, including construction of cell membranes, regulation of cell signaling, and energy storage. However, visualizing lipids using conventional histology is difficult because of their simple structure and variety of molecular species.

Several reports have shown that IMS can be used for imaging of localization of lipids, such as phospholipids (PLs), neutral lipids (NLs), and sphingolipids (SPLs) [6–9]. In particular, Fourier transform ion cyclotron resonance-mass spectrometry (FTICR-MS) was used to specifically identify the exact mass and isotopic distribution of target molecules using ultra-high-mass resolution (>500,000) and structural analysis with an MS/MS approach [10, 11]. Thus, MALDI-FTICR-IMS can clearly visualize several target molecules as independent images [10, 12].

SPLs, including sphingomyelin (SM), are signaling regulators and components of lipid microdomains [13, 14]. SPLs contain ceramide (Cer) as a backbone, in which long chain fatty acid (LCFA) or very long chain fatty acid (VLCFA) is amide-linked to the sphingoid base. The synthesis of Cers, the precursor of SMs and glucosylceramides (GlcCers), is mediated by six isoforms of Cer synthase (CerS) and dihydroceramide desaturase 1 [15]. Each CerS isoform has substrate specificity for the lengths of acyl-CoA [15]. Part of the Cers is transferred from the endoplasmic reticulum to the Golgi apparatus by a Cer transport protein, and is then converted to SM by the transfer of phosphocholine from phosphatidylcholine through SM synthase (SMS) [16, 17]. We and others have independently reported that the distribution of SMs in the mouse brain differed with the lengths of their acyl-chain [12, 18]. For example, C18-SMs were localized in cell body-rich gray matter, while C24-SMs were located in myelin-rich white matter. In addition, the specific distribution of SMs was regulated by the expression of CerS isoforms, CerS1 (which produce C18-Cer) and CerS2 (which produce C22- and C24-Cers) [12]. Thus, the lengths of the acyl-chain in SMs are important factors that regulate their molecular function in the brain.

The kidneys play essential physiological roles in the body, including regulating the electrolyte balance, blood filtering, and removing urea and creatinine (Cre). A number of SPLs have been reported to be involved in renal function and disease. Various sulfatides, a class of amphiphilic anionic glycosphingolipids (GSLs), also play an essential role in the ammonia exchange process [19]. Fabry disease is a genetically inherited X-linked lysosomal storage disease caused by a deficiency in α-galactosidase A (α-Gal A) activity, which causes accumulation of globotriaosylceramide (Gb3) in blood vessels, kidneys, and heart [20]. Using IMS, accumulation of individual Gb3 was reported in the renal Cx of the kidneys in α-Gal A-knockout (KO) mice [21].

The kidneys also contain a high concentration of SMs compared with the brain and liver [22]. Using IMS in the rat kidneys, it was reported that C16-SMs were distributed in the renal cortex (Cx) and concentrated in the border of the Cx-medulla (Med), while C24-SMs and Cers were distributed in the renal Cx [23]. Thus, SMs likely play important roles in kidney structure and function. However, the regulatory mechanism of region-specific SM metabolism in the kidney remains unclear.

The *de novo* synthesis of SM is dependent on SMS1 and 2 in the Golgi apparatus or SMS2 in the plasma membranes [16, 17]. The majority of studies have examined SMS2 in the liver, as it is the major isoform and determinant of SM in the liver [24–26]. We also reported that genetic inhibition of SMS2 ameliorates diet-induced obesity, fatty liver disease, and insulin resistance [27]. Further, SMS2 is highly expressed in the kidneys, similarly to the liver and gastrointestinal organs [22]. However, the contribution of SMS2 in the metabolism of SPLs in the kidneys is not fully understood.

Thus, the aim of this study was to examine the effect of SMS2 deficiency on SPLs metabolism in the kidneys versus liver of mice fed with a normal diet (ND) or a high fat diet (HFD) using a quantitative and imaging lipidomic approach.

## Materials and Reagents

High-performance liquid chromatography-grade methanol, acetonitrile, chloroform, n-hexane, and 2-propanol were purchased from Kanto Chemical Co., Inc. (Tokyo, Japan). Trifluoroacetic acid, ammonium acetate, potassium acetate, potassium hydroxide, and hydrochloric acid were obtained from Wako Pure Chemical Co., Ltd. (Osaka, Japan). 2,5-dihydroxybenzoic acid (DHB) and formic acid were purchased from Sigma-Aldrich (Saint Louis, MO). Indium tin oxide (ITO) glass slides were obtained from Bruker Daltonics (Bremen, Germany).

SM (d18:1/16:0), SM (d18:1/18:0), SM (d18:1/18:1), SM (d18:1/24:0), SM (d18:1/24:1), SM (d18:1/16:0-d31), Cer (d18:1/16:0), Cer (d18:1/18:0), Cer (d18:1/18:1), Cer (d18:1/22:0), Cer (d18:1/24:0), Cer (d18:1/24:1), and Cer (d18:1/16:0-d31) standards were purchased from Avanti-Polar Lipids Inc. (Alabaster, AL). Trizol RNA Separation Reagent was obtained from Life Technologies (Carlsbad, CA). The RNeasy Mini Kit was purchased from Qiagen (Venlo, the Netherlands). PrimeScript RT Reagent Kit and SYBR Premix Ex Taq II and Perfect Real Time Primer were obtained from Takara Bio Inc. (Otsu, Japan). Creatinine (urinary) Colorimetric Assay Kit was purchased from Cayman Chemical Company (Ann Arbor, MI). Mouse Albumin ELISA Kit was purchased from Shibayagi Co., Ltd. (Shibukawa, Japan).

### Animals

All animal experiments were approved by the Animal Care and Use Committee of the Graduate School of Medicine, Hokkaido University (Sapporo, Japan). The protocol conformed to the *Guide for the Care and Use of Laboratory Animals* (US National Institutes of Health, Bethesda, MD). SMS2-KO mice were generated as described previously on a C57BL/6N genetic background [27]. The same genetic background WT mice were used as controls. Mice were housed under controlled temperature and humidity with a 12 h light–dark cycle. Before the experiments, mice were kept for 1 week for acclimatization. Male WT and SMS2-KO mice were fed with a ND (4.6% fat, CE-2; CLEA Japan Inc., Tokyo, Japan) or a HFD (34.9% fat, 58Y1; TestDiet, St. Louis, MO) for 3 weeks beginning at 7 weeks of age. Foods and water were provided *ad libitum*. Tissues were removed from mice at 10 weeks of age under isoflurane anesthesia after 16 h of fasting. Tissues were weighed and snap-frozen on dry ice, and stored at -80°C until analysis.

### Lipid extraction and lipid measurement by liquid chromatography/electrospray ionization-tandem mass spectrometry (LC/ESI-MS/MS)

Lipid extraction for lipid measurement was performed as described previously [12]. LC/ESI-MS/MS analyses were performed using an UltiMate 3000 HPLC (Thermo Fisher Scientific Inc., Waltham, MA) and a Q Exactive Plus Hybrid Quadrupole-Orbitrap Mass Spectrometer (Thermo Fisher Scientific Inc.) as described previously [12]. The internal standards used were stable isotopes of SM and Cer, SM (d18:1/16:0-d31) and Cer (d18:1/16:0-d31), respectively. Lipid identification was performed using Lipid Search software (Mitsui Knowledge Industry Co., Ltd., Tokyo, Japan). SPL levels in extracts were quantified with standards of each lipid by Xcalibur processing and instrument control software (Thermo Fisher Scientific Inc.).

### MALDI-FTICR-IMS

MALDI-FTICR-IMS analyses were performed as described previously [12]. Briefly, frozen tissues were sectioned at 10 μm thick and mounted on ITO glass slides. Each concentration of SM standards was also mounted on ITO glass slides and dried. A DHB solution (30 mg/mL DHB, 20 mM potassium acetate, 50% MeOH, 0.2% trifluoroacetic acid) was sprayed onto tissue sections or standards mounted on ITO glass slides using Image Prep (Bruker Daltonics). IMS analyses were performed using a 7T solariX XR system (Bruker Daltonics). IMS conditions were described previously [12]. The target peak obtained was accumulated and split by collision-induced dissociation (CID)-fragmentation to obtain structural information. Mass spectra were analyzed and obtained using Data Analysis v4.3 (Bruker Daltonics). FlexImaging v4.0 (Bruker Daltonics) was used for data processing and image generation. Imaging data were normalized by the root means square method.

### RNA extraction and real-time polymerase chain reaction (PCR)

Total RNA was extracted using Trizol RNA Separation Reagent and an RNeasy Mini Kit according to the manufacturer’s protocols. Reverse transcription was performed using a PrimeScript RT Reagent Kit and PCR Thermal Cycler SP (Takara Bio Inc.). Real-time PCR was performed using SYBR Premix Ex Taq II, Perfect Real Time Primers (S1 Table), and a 7500 Real-time PCR system (Life Technologies). Expression of *18S* rRNA or *Gapdh* was used as the endogenous reference for each sample. The relative expression of each gene was expressed as a value relative to its expression in WT mice fed with a ND. The relative expression of each gene was also expressed as a value relative to expression of *Sgms1* in WT mice.

### Histology

The morphology of the liver and kidney tissue was observed by hematoxylin and eosin (H&E) staining of the serial frozen sections as described previously [12].

### Urinary albumin (Alb)/Cre measurements

Spot urine samples were collected from mice by briefly restraining each mouse, resulting in spontaneous urination on a Petri dish. Urinary Alb and Cre concentrations were measured using Creatinine (urinary) Colorimetric Assay Kit and Mouse Albumin ELISA Kit according to the manufacturer’s protocol.

### Statistical analyses

Experimental values are presented as mean ± SEM. Statistical analyses were performed by two-way ANOVA, followed by *post hoc* Tukey-Kramer test for multiple group comparisons, using SAS v9.4 (SAS Institute, Cary, NC). *p* < 0.05 was considered statistically significant.

## Results

### Detection and quantification of SMs using standards

**Fig 1:**
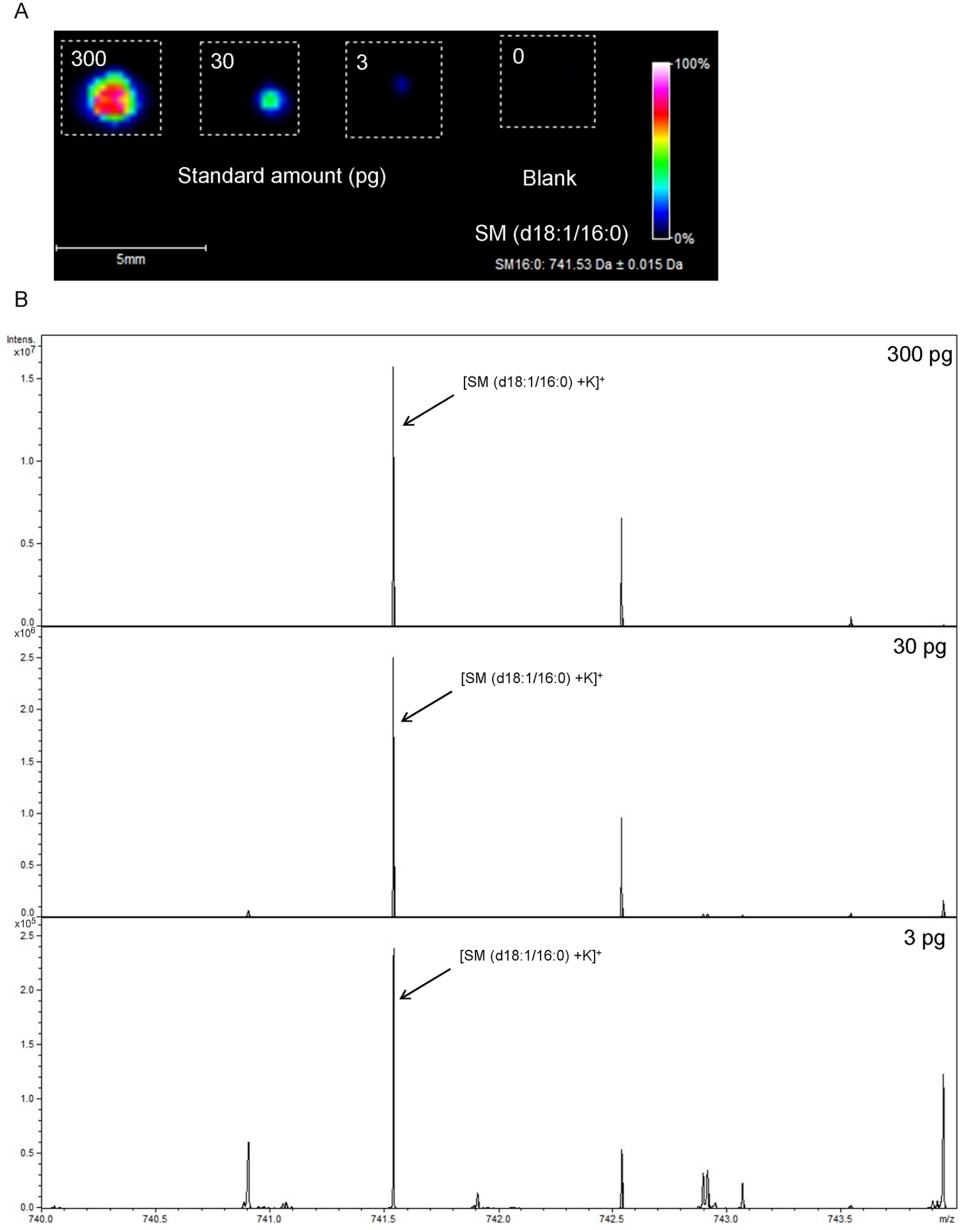
Analysis of SM (d18:1/16:0) standard by MALDI-FTICR-IMS. (A) Visualization of [SM (d18:1/16:0) +K]^+^ at a range from 3–300 pg on an ITO glass slide. (B) Obtained mass spectra of [SM (d18:1/16:0) +K]^+^ (indicated with arrows) at a range from 3–300 pg on ITO glass slides. Scale bar = 5 mm. Data were obtained from three individual samples.

To demonstrate the potential of MALDI-FTICR-IMS for the detection and quantitation of SMs, we first confirmed the *m/z* of each SM species using standards; i.e., SM (d18:1/16:0), SM (d18:1/18:0), SM (d18:1/18:1), SM (d18:1/24:0), and SM (d18:1/24:1) on ITO glass slides using the positive ion detection mode of MALDI-FTICR-IMS. SM (d18:1/16:0), SM (d18:1/18:0), SM (d18:1/18:1), SM (d18:1/24:0), and SM (d18:1/24:1) standards were detected as potassium adducts with an observed *m/z* of 741.5378, 769.5671, 767.5516, 853.6629, and 851.6529, respectively (Table 1). Next, we investigated the relationship between the intensity and concentration of each SM standard on ITO glass slides using the positive ion detection mode of MALDI-FTICR-IMS. We set regions of interest (ROIs) around each amount of the SM standard spot and measured the average intensities of SM (d18:1/16:0), *m/z* 741.54 (Fig 1A and 1B). There was a good correlation between the intensity and amount of SM standard at the range from 3–300 pg (Fig 1A and 1B). The analysis of other SM standards, SM (d18:1/18:0), SM (d18:1/18:1), SM (d18:1/24:0), and SM (d18:1/24:1), also showed a good correlation between the intensities and amounts of SM standards at the range from 3–300 pg (Table 1).

**Table 1:** Summary of quantitative data obtained from each SM standard by MALDI-FTICR-IMS.

		Averaged Intensities			
SM species	Observed *m/z*	300 pg	30 pg	3 pg	R^2^
d18:1/16:0	741.5378	26652416±6053895	2936522±399269	283597±131599	0.9999
d18:1/18:1	767.5516	63949312±7827226	5269675±551402	488060±165657	0.9998
d18:1/18:0	769.5671	38218069±5675980	4038837±1173243	570715±171020	1.0000
d18:1/24:1	851.6529	38946688±14297676	5925120±541362	1011664±225944	0.9986
d18:1/24:0	853.6629	43828736±11142832	7990144±1320577	1359525±212954	0.9960

Obtained *m/z*, averaged intensities, and R^2^ of each SM standard are shown. Data were obtained from three individual samples (mean ± SD).

### Detection and characterization of SMs in tissue sections

Next, we evaluated mass spectra in the range 500 < *m/z* < 900 from liver and kidney sections of mice fed with a ND using MALDI-FTICR-IMS (S1 and S2 Figs). Five mass peaks, *m/z* 741.5293, 769.5547, 825.6199, 851.6350, and 853.6502 were assigned to SM (d18:1/16:0), SM (d18:1/18:0), SM (d18:1/22:0), SM (d18:1/24:1), and SM (d18:1/24:0), respectively, as potassium adducts using their exact mass values (S1 and S2 Figs, Table 2). These assignments of SM were also confirmed with structural analyses of each peak by CID fragmentation in each tissue (Table 2). Phosphocholine was confirmed by neutral losses of 59 u and 183 u from precursor ions, as for our previous report in the mouse brain [12]. Although distinguishing between molecules with exactly the same mass and product ions was impossible, it was reported that the sphingoid base of SMs was d18:1, in contrast to both the d18:1 and d20:1 sphingoid bases observed in gangliosides [28]. Thus, five peaks were assigned to SMs as shown in Table 2.

**Table 2:** Summary of SM molecular species obtained from the liver and kidney sections by MALDI-FTICR-IMS.

SM species	Adduct ion	Parent ions *m/z*	Product Ions *m/z*	
d18:1/16:0	[M+K]^+^	741.5293	682.4419	558.4597
d18:1/18:0	[M+K]^+^	769.5547	710.4703	586.4889
d18:1/22:0	[M+K]^+^	825.6199	766.4622	642.4801
d18:1/24:1	[M+K]^+^	851.6350	792.5502	668.5641
d18:1/24:0	[M+K]^+^	853.6502	794.5677	670.5160

Obtained *m/z* of parent and product ions in the tissue sections are shown. Data were obtained from two individual samples.

### Localization of SMs in the liver sections

We next visualized the distribution pattern of SM (d18:1/16:0; *m/z* 741.53), SM (d18:1/18:0; *m/z* 769.55), SM (d18:1/22:0; *m/z* 825.62), SM (d18:1/24:1; *m/z* 851.64), and SM (d18:1/24:0; *m/z* 853.65) in the liver sections (S1 and S3 Figs). The intensities of SM (d18:1/22:0), SM (d18:1/24:0), and SM (d18:1/24:1) were detected strongly in the liver section by MALDI-FTICR-IMS, while those of SM (d18:1/16:0), SM (d18:1/18:0), and SM (d18:1/18:1) were low (S3 Fig). Whereas there was no specific distribution of SMs in the liver, which is considered a homogeneous tissue, strong intensities of SM (d18:1/16:0) and SM (d18:1/18:0) were observed in the region of the vessels (S3A–S3C Fig).

### Localization of SMs in the kidney sections

**Fig 2:**
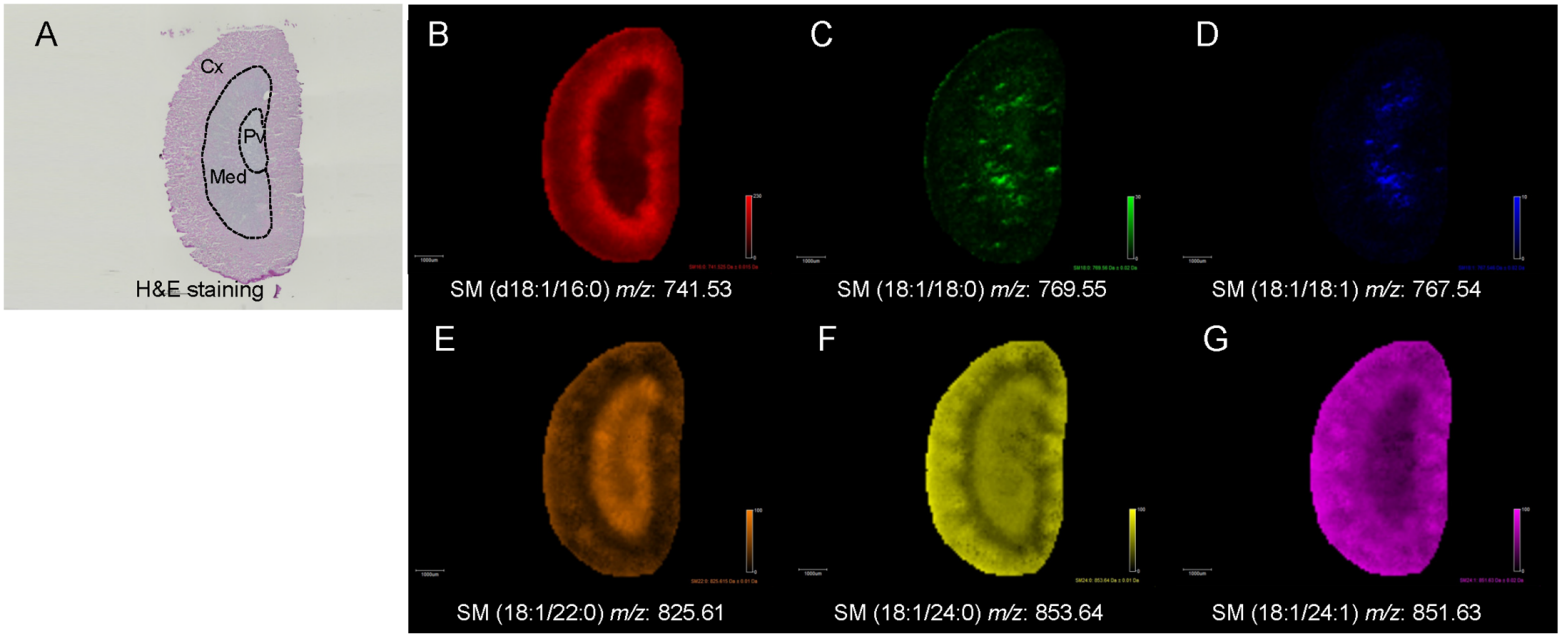
Localization of SM molecular species in the kidney sections of WT mice fed with a ND. (A) Representative image of a kidney section stained with H&E. Renal Cx, Med, and pelvis (Pv) are divided with the broken lines. Representative images of (B) [SM (d18:1/16:0) +K]^+^, (C) [SM (d18:1/18:0) +K]^+^, (D) [SM (d18:1/18:1) +K]^+^, (E) [SM (d18:1/22:0) +K]^+^, (F) [SM (d18:1/24:0) +K]^+^, and (G) [SM (d18:1/24:1) +K]^+^ are shown. Scale bar = 1 mm. Data were obtained from two individuals.

We also visualized the distribution pattern of SM (d18:1/16:0; *m/z* 741.53), SM (d18:1/18:0; *m/z* 769.55), SM (d18:1/22:0; *m/z* 825.62), SM (d18:1/24:1; *m/z* 851.64), and SM (d18:1/24:0; *m/z* 853.65) in the kidney sections (Fig 2 and S2 Fig). The intensities of SM (d18:1/16:0), SM (d18:1/22:0), SM (d18:1/24:0), and SM (d18:1/24:1) were detected strongly in the kidney sections by MALDI-FTICR-IMS, while those of SM (d18:1/18:0) and SM (d18:1/18:1) were low (Fig 2). Interestingly, a unique distribution pattern of SM molecular species was observed in the kidney section (Fig 2). SM (d18:1/16:0) was localized mainly in the region of the arcuate vein and border of the renal Cx and Med (Fig 2A and 2B). By contrast, SM (d18:1/22:0) was localized specifically in the renal Med (Fig 2A and 2E). SM (d18:1/24:0) was localized in both the renal Cx and Med (Fig 2A and 2F), while SM (d18:1/24:1) was found in the renal Cx (Fig 2A and 2G).

### Quantitative visualization of SM localization in the liver sections of WT and SMS2-KO mice fed with a ND or a HFD by LC/ESI-MS/MS and MALDI-FTICR-IMS

As the functions of SMs have been well studied in the liver, we first measured the SMs contents in the liver extracts of WT and SMS2-KO mice fed with a ND or a HFD by LC/ESI-MS/MS (S4A Fig). Three weeks of HFD-feeding markedly altered the profile of SMs in the liver, with an increase in C18–C22-SMs and a decrease in C24-SMs (S4A Fig). In mice fed with a ND, SMS2 deficiency primarily decreased C22- and C24-SMs (S4A Fig). The effects of SMS2 deficiency on SM metabolism were larger in mice fed with a HFD compared with mice fed with a ND. SMS2 deficiency significantly decreased C18–C24-SMs in mice fed with a HFD (S4A Fig).

To clarify that MALDI-FTICR-IMS allowed quantitative visualization of the tissue distribution of SM molecular species, we next visualized the localization of SMs in the liver sections of WT and SMS2-KO mice fed with a ND or a HFD by MALDI-FTICR-IMS (S4B–S4G Fig). The intensities of SM (d18:1/16:0) and SM (d18:1/18:0) were low in the liver sections of all groups of mice (S4C and S4D Fig). In the liver sections of WT mice, SM (d18:1/22:0) was markedly increased by HFD-feeding, while the C24-SMs, SM (d18:1/24:0) and SM (d18:1/24:1), were decreased, as observed for LC/ESI-MS/MS data (S4E–S4G Fig). Regardless of feeding condition, SMS2 deficiency decreased C22- and C24-SMs in the liver sections (S4E–S4G Fig). Because of their low intensities, the effects of HFD-feeding on these SM molecules could not be observed in the liver sections of SMS2-KO mice (S4E–S4G Fig).

### Evaluation of SPL metabolism in the kidneys of WT and SMS2-KO mice fed with a ND or a HFD by LC/ESI-MS/MS

**Fig 3:**
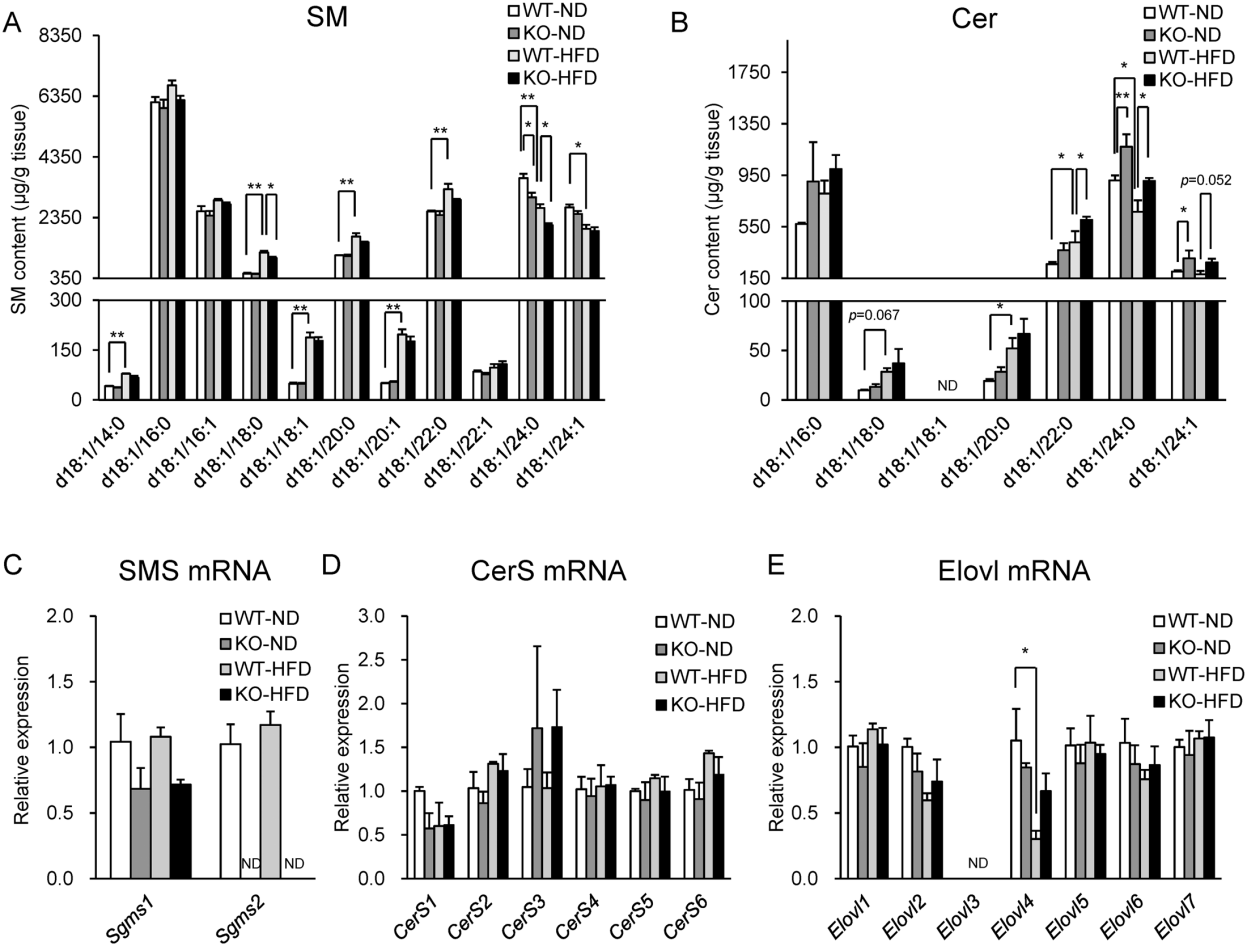
Effects of SMS2 deficiency on SPLs metabolism in the liver of mice fed with a ND or a HFD. Measurement by LC/ESI-MS/MS of (A) SMs and (B) Cers in whole-kidney extracts of mice fed with a ND or a HFD. The levels of SMs and Cers were normalized with internal standards, SM (d18:1/16:0-d31) and Cer (d18:1/16:0-d31), and expressed as μg per g tissue. The expression of (C) SMS, (D) CerS, and (E) Elovl mRNA in whole-kidney extracts. Expression of *18S* rRNA was used as the endogenous reference for each sample. The expression level of each gene was shown as a value relative to that in WT mice fed with a ND. Data are means ± SEM; n = 3 per group. Significant differences compared with a corresponding value in WT mice are shown. *p < 0.05, **p < 0.01, two-way ANOVA followed by the *post hoc* Tukey-Kramer test.

To demonstrate the effects of HFD-feeding and SMS2 deficiency on SPL metabolism in the kidneys, we next measured the contents of SMs and Cers in the whole-kidney extracts of WT and SMS2-KO mice fed with a ND or a HFD by LC/ESI-MS/MS (Fig 3). C18–C22-SMs and Cers were increased, while C24-SMs and Cers were decreased, by three weeks of HFD-feeding, as observed for the liver (Fig 3A and 3B). In mice fed with a ND, SMS2 deficiency primarily decreased C24-SMs, but increased C24-Cers (Fig 3A and 3B). SMS2 deficiency showed a trend for decreased C18–C24-SMs in the kidneys of mice fed with a HFD, but increased C22–C24-Cers (Fig 3A and 3B). To examine the mechanism of the acyl-chain specific alterations in SPLs by HFD-feeding or SMS2 deficiency, we measured the gene expression of enzymes relating to SPL metabolism (e.g., SMS, CerS, and the fatty acid elongase (Elovl) family) in the kidneys of WT and SMS2-KO mice fed with a ND or a HFD (Fig 3C–3E). However, there were no marked changes in the expression of these genes by HFD-feeding and SMS2 deficiency, except for the expression of *Elovl4* in mice fed with a HFD (Fig 3C–3E).

### Analysis of SM localization in the kidney sections of WT and SMS2-KO mice fed with a ND or a HFD by MALDI-FTICR-IMS

**Fig 4:**
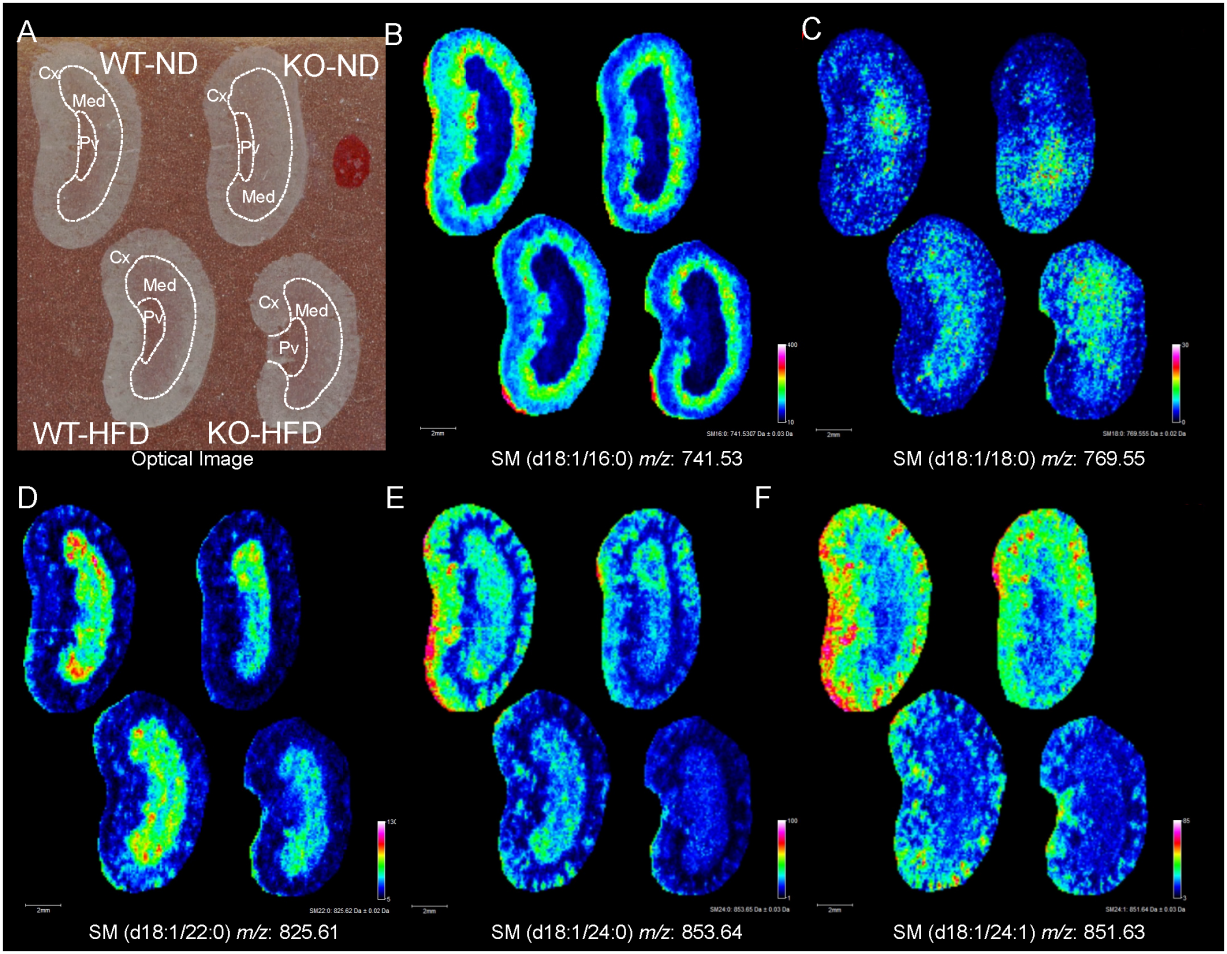
Visualization of SM molecular species in the kidney sections of WT and SMS2-KO mice fed with a ND or a HFD. (A) Representative optical images of kidney section obtained from each group of mice. Renal Cx, Med, and Pv are shown with broken lines. Representative images of (B) [SM (d18:1/16:0) +K]^+^, (C) [SM (d18:1/18:0) +K]^+^, (D) [SM (d18:1/22:0) +K]^+^, (E) [SM (d18:1/24:0) +K]^+^, and (F) [SM (d18:1/24:1) +K]^+^ are shown. Scale bar = 2 mm. Data were obtained from three individuals per group.

We also visualized the localization of SMs in the kidney sections of WT and SMS2-KO mice fed with a ND or a HFD by MALDI-FTICR-IMS. SM (d18:1/16:0) was mainly distributed in the border of the renal Cx and Med in all groups of mice (Fig 4B). Although we focused on the border of the renal Cx and Med, there were no observed changes in SM (d18:1/16:0) by HFD-feeding and SMS2 deficiency (Fig 4B). Because the intensity of SM (d18:1/18:0) was very low in the kidney sections, the effects of HFD-feeding and SMS2 deficiency could not be evaluated in this SM species (Fig 4C). MALDI-FTICR-IMS revealed that SM (d18:1/22:0) was highly distributed in the renal Med of WT mice and predominantly decreased by the genetic inhibition of SMS2 (Fig 4D), although the reduction was small in the analyses of whole-kidney extract by LC/ESI-MS/MS (Fig 3A). SM (d18:1/24:0) and SM (d18:1/24:1) were decreased in the renal Cx, where these SMs were predominantly distributed, by HFD-feeding and SMS2 deficiency (Fig 4E and 4F).

## Discussion

In the present study, we investigated the effects of SMS2 deficiency on the distribution and concentration of SMs in the liver and kidneys of mice fed with a ND or a HFD by combination analysis of MALDI-FTICR-IMS and LC/ESI-MS/MS. MALDI-FTICR-IMS analysis demonstrated a region-specific reduction of SMs depending on the lengths of acyl-chain in the kidneys of SMS2 deficient mice.

Although IMS can provide quantitative information on endogenous lipids using tissue homogenates with addition of a stable isotope [29], there are limited data on the potential of IMS for quantitative analysis of lipids. Thus, we first confirmed the *m*/*z* and relationship between the intensity and concentration of SM standards using MALDI-FTICR-MS. Each SM standard was detected as a potassium adduct, probably due to potassium acetate in DHB solution [6, 12]. We also found good correlations between the mass peak intensity and concentration of each SM standard. Thus, our data suggest that MALDI-FTICR-IMS should allow visualization of tissue distribution of target molecules with quantitative manner.

Next, we examined the SM content and localization in the liver and kidney sections using MALDI-FTICR-IMS. Each SM candidate was detected as a potassium adduct in the tissue sections. We also performed CID-fragmentation of each SM candidate on the tissue sections, and confirmed that each of them contained a phosphocholine. MALDI-FTICR-MS could not determine the lengths of the sphingoid base and acyl-chain, whereas LC/ESI-MS/MS demonstrated that d18:1 sphingosine was a major sphingoid base of SMs, as previously described [12]. Thus, we determined each SM by its exact mass and neutral loss of the phosphocholine. Further, we visualized these identified SMs in the liver and kidney sections of mice fed with a ND. In the liver, the SM species, SM (d18:1/16:0) and SM (d18:1/18:0), were highly detected in the region of blood vessels, which likely reflected SMs in the blood. SM (d18:1/16:0) was localized mainly in the region of the arcuate vein and the border of the renal Cx and Med. By contrast, SM (d18:1/22:0) was localized specifically in the renal Med. SM (d18:1/24:0) was localized in both the renal Cx and Med, while SM (d18:1/24:1) was localized in the renal Cx. Muller et al. demonstrated similar findings in rat kidney sections using silver nanoparticle as a matrix and MALDI-LTQ-Orbitrap [23]. Thus, these data suggest that the molecular functions of SM may be regulated according to the lengths of acyl-chains in the kidney over various animal species.

Obesity-related ectopic lipid accumulation can cause organ injury, including the liver and kidneys [30]. White adipose tissue (WAT)-derived free fatty acid (FFA) and diacylglycerol (DG) are key factors involved in insulin resistance, fatty liver disease, and kidney dysfunction [30]. SPLs such as SM and Cer also contribute to hepatic insulin resistance and fatty liver disease [26, 27, 31]. It was also reported that the concentrations of Cer and sphingosine in the kidneys were altered during ischemic acute renal failure [32, 33]. Further, HFD-induced kidney damage is partly caused by excessive accumulation of Cer [34]. Recent studies have reported differences in the contributions of SPLs of different acyl-chain lengths to hepatic insulin resistance and fatty liver disease. Prior analyses of CerS6-KO mice demonstrated that C16-Cer promotes HFD-induced hepatic insulin resistance and fatty liver disease [35]. By contrast, it was postulated that C24-Cer positively regulates insulin signaling and inhibits fatty acid uptake in hepatocytes when examined in CerS2-KO mice *in vivo* or *in vitro* [36, 37]. However, the relationship between the length of acyl-chain in SPLs and renal dysfunction remains unclear. As observed in the liver, using LC/ESI-MS/MS and MALDI-FTICR-IMS we found that HFD-feeding increased C18–C22 SMs and Cers, but decreased C24-SM and Cer, in the kidneys. Thus, HFD-induced alteration in the SPL profiles may also contribute to kidney damage.

The expression of the *CerS* and *Elovl* genes in the kidney could not explain how HFD-feeding caused the specific alterations in the profiles of SMs and Cers due to their acyl-chain lengths. HFD was previously reported to contain lower concentrations of C24:0 and C24:1 fatty acids compared with ND [38]. Therefore, the reduction of C24-SMs and Cers in the kidneys of mice fed with a HFD may depend on the fatty acid composition in the diet.

SMS2 is a key enzyme involved in *de novo* synthesis of SM in plasma membranes [16, 17]. Whereas the contribution of SMS2 on SPL metabolism is well studied in the liver, we demonstrated that SMS2 is highly expressed in the kidneys, similar to the liver and gastrointestinal organs (S5 Fig). We measured the content of SMs and Cers in the whole-kidney extracts of WT and SMS2-KO mice fed with a ND or a HFD by LC/ESI-MS/MS. The effect of SMS2 deficiency on SPLs metabolism in the kidneys was smaller than that in the liver. For example, a trend for decreased C18–C24-SMs was observed in the kidneys of SMS2-KO mice. To the best of our knowledge, there have been no reports that show the contribution of SMS1 to SPL metabolism in the kidneys. However, we confirmed that the expression of *Sgms1* was lower than that of *Sgms2* in the kidneys. Thus, the contribution of SMS1 may be equal to or smaller than that of SMS2 to *de novo* SM synthesis in the kidneys. We also visualized the localization of SMs in the kidney sections by MALDI-FTICR-IMS. SM (d18:1/22:0) was mainly decreased in the renal Med by SMS2 deficiency. By contrast, SM (d18:1/24:0) and SM (d18:1/24:1) were predominantly decreased in the renal Cx with SMS2 deficiency. Thus, MALDI-FTICR-IMS was able to reveal the contribution of SMS2 to region-specific SM metabolism in the kidneys, which was unable to be determined in analysis of whole-kidney extract by LC/ESI-MS/MS. Therefore, SMS2 is likely responsible for the region-specific *de novo* synthesis of SM in the kidneys.

The function of SMs in the kidney is unclear, as severe phenotypes have not been observed in the kidneys of CerS2- and CerS6-KO mice [39, 40]. In the kidneys of CerS2-KO mice, VLCFA-containing SMs were markedly reduced to very low levels. However, total SM levels were maintained by alternative upregulation of C16-SM [39]. By contrast, whereas C16-SM was strongly reduced, total SM levels were maintained by the mild up-regulation of VLCFA-containing SMs in the kidneys of CerS6-KO mice [40]. Thus, these compensation mechanisms of SMs would maintain the function and structure of the kidneys in these mice. However, in the present study the distribution of SMs differed markedly, due to their acyl-chain lengths. Future histological analyses of lipids are required to clarify the function of SMs in the kidneys.

Although several groups have studied the phenotype of SMS2-KO mice [24–27], there are no reports that show any kidney-related phenotype of SMS2-KO mice. Thus, we demonstrated the effects of SMS2 deficiency on the HFD-induced renal injury in this study (S6 Fig). While long-term HFD-feeding significantly increased the urinary Alb/Cre ratio, as previously described [34], SMS2 deficiency did not change the ratio in mice fed with a ND or a HFD (S6 Fig). Because it has been stated that renal injury by HFD-feeding is mild [41], the effects of SMS2 deficiency should be studied using other models of kidney injury to clarify the function of SMS2 in the kidneys.

In summary, this is the first study to report the contribution of SMS2 on acyl-chain- and region-specific SM metabolism in the kidneys of mice fed with a ND or a HFD.

## Supporting Information

S1 Fig Representative mass spectra of SM molecular species in liver sections of ND-fed WT mice. Representative mass spectra at range from *m/z* 739 to *m/z* 854 obtained from mouse liver sections by MALDI-FTICR-MS. Arrows indicate the peaks of [SM (d18:1/16:0) +K]^+^, [SM (d18:1/18:0) +K]^+^, [SM (d18:1/22:0) +K]^+^, [SM (d18:1/24:0) +K]^+^, and [SM (d18:1/24:1) +K]^+^. Data were obtained from two individuals.
(TIFF)

S2 Fig Representative mass spectra of SM molecular species in kidney sections of ND-fed WT mice. Representative mass spectra at range from *m/z* 739 to *m/z* 854 obtained from mouse kidney sections by MALDI-FTICR-MS. Arrows indicate the peaks of [SM (d18:1/16:0) +K]^+^, [SM (d18:1/18:0) +K]^+^, [SM (d18:1/22:0) +K]^+^, [SM (d18:1/24:0) +K]^+^, and [SM (d18:1/24:1) +K]^+^. Data were obtained from two individuals.
(TIFF)

S3 Fig Localization of SM molecular species in the liver sections of WT mice fed with a ND. (A) Representative image of a liver section with H&E staining. Arrows indicate the region of the vessels. Representative images of (B) [SM (d18:1/16:0) +K]^+^, (C) [SM (d18:1/18:0) +K]^+^, (D) [SM (d18:1/18:1) +K]^+^, (E) [SM (d18:1/22:0) +K]^+^, (F) [SM (d18:1/24:0) +K]^+^, and (G) [SM (d18:1/24:1) +K]^+^ are shown. Scale bar = 1 mm. Data were obtained from two individuals.
(TIFF)

S4 Fig Quantification and visualization of SM molecular species in the liver of WT and SMS2-KO mice fed with a ND or a HFD. (A) Measurement by LC/ESI-MS/MS of SMs in the liver extracts of mice fed with a ND or a HFD. The levels of SMs were normalized with internal standards, SM (d18:1/16:0-d31), and expressed as μg per g tissue. Data are means ± SEM; n = 3 per group. Significant differences compared with a corresponding value in WT mice are shown. *p < 0.05, ** p < 0.01, two-way ANOVA followed by *post hoc* Tukey-Kramer test. (B) Representative optical images of liver sections obtained from each group of mice. Arrows indicate the region of the vessels. Representative images of (C) [SM (d18:1/16:0) +K]^+^, (D) [SM (d18:1/18:0) +K]^+^, (E) [SM (d18:1/22:0) +K]^+^, (F) [SM (d18:1/24:0) +K]^+^, and (G) [SM (d18:1/24:1) +K]^+^ are shown. Scale bar = 2 mm. Data were obtained from three individuals per group.
(TIFF)

S5 Fig Relative expression of *Sgms1* and *Sgms2* in organs of WT and SMS2-KO mice. Expression of *Gapdh* was used as the endogenous reference for each sample. Each gene was expressed as a value relative to its expression of *Sgms1* in WT mice. Data are mean ± SEM; n = 3 per group.
(TIFF)

S6 Fig Urinary Alb/Cre in WT and SMS2-KO mice fed with a ND or a HFD for 21 weeks. Data are mean ± SEM; n = 8–12 per group. Significant differences compared with a corresponding value in WT mice are shown. *p < 0.05, **p < 0.01, two-way ANOVA followed by *post hoc* Tukey-Kramer test.
(TIFF)

S1 Table Summary of primer sets for quantitative real-time PCR. (DOCX)
