# Supplementary figures and images for: Imaging Mass Spectrometry Reveals Acyl-Chain- and Region-Specific Sphingolipid Metabolism in the Kidneys of Sphingomyelin Synthase 2-Deficient Mice
Source: PLoS One. 2016 Mar 24;11(3):e0152191. doi: 10.1371/journal.pone.0152191 (PMC4806983; doi:10.1371/journal.pone.0152191)

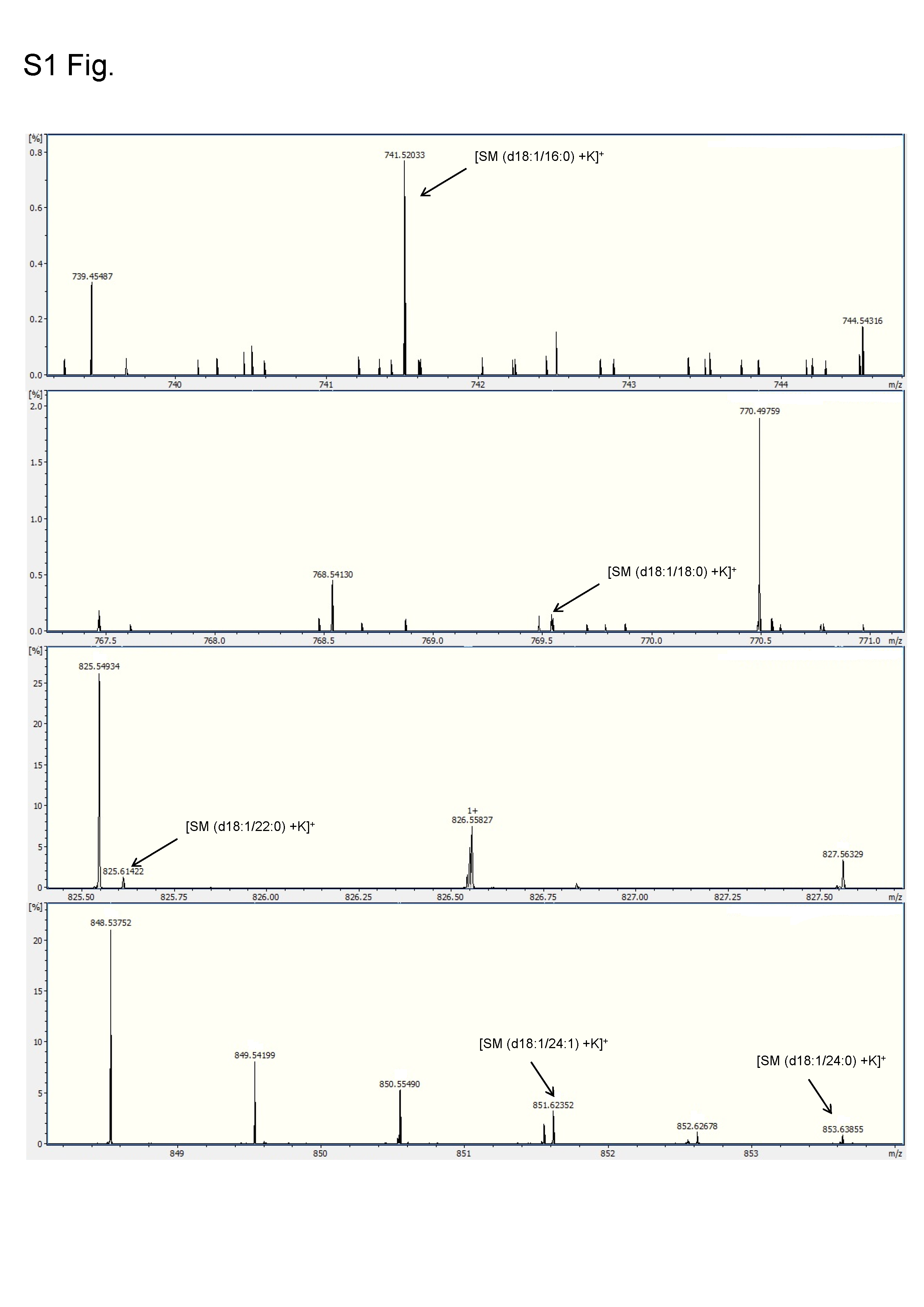

Supplement: S1 Fig — Representative mass spectra at range from m/z 739 to m/z 854 obtained from mouse liver sections by MALDI-FTICR-MS. Arrows indicate the peaks of [SM (d18:1/16:0) +K]+, [SM (d18:1/18:0) +K]+, [SM (d18:1/22:0) +K]+, [SM (d18:1/24:0) +K]+, and [SM (d18:1/24:1) +K]+. Data were obtained from two individuals. (TIFF) [file pone.0152191.s001.tiff]

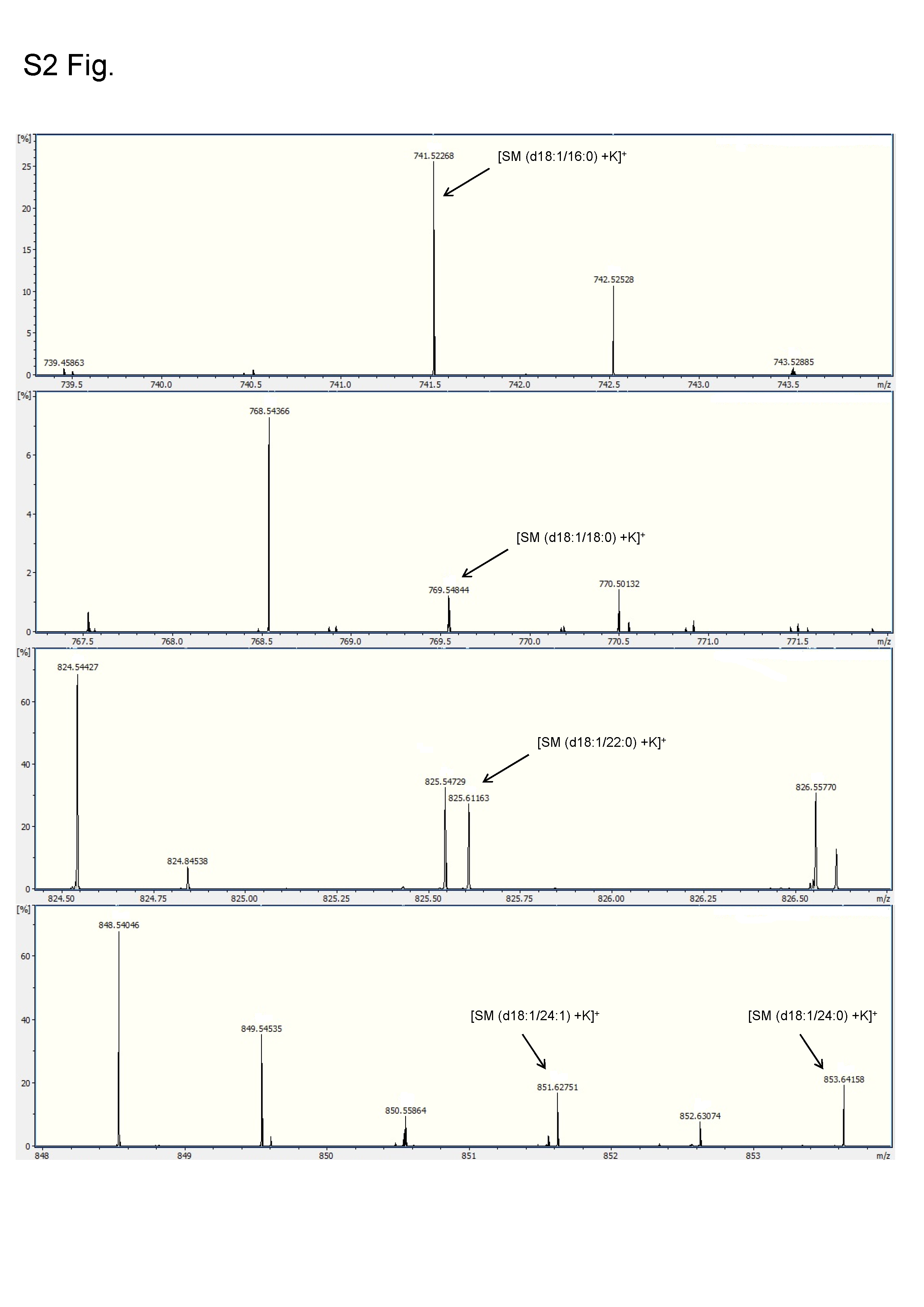

Supplement: S2 Fig — Representative mass spectra at range from m/z 739 to m/z 854 obtained from mouse kidney sections by MALDI-FTICR-MS. Arrows indicate the peaks of [SM (d18:1/16:0) +K]+, [SM (d18:1/18:0) +K]+, [SM (d18:1/22:0) +K]+, [SM (d18:1/24:0) +K]+, and [SM (d18:1/24:1) +K]+. Data were obtained from two individuals. (TIFF) [file pone.0152191.s002.tiff]

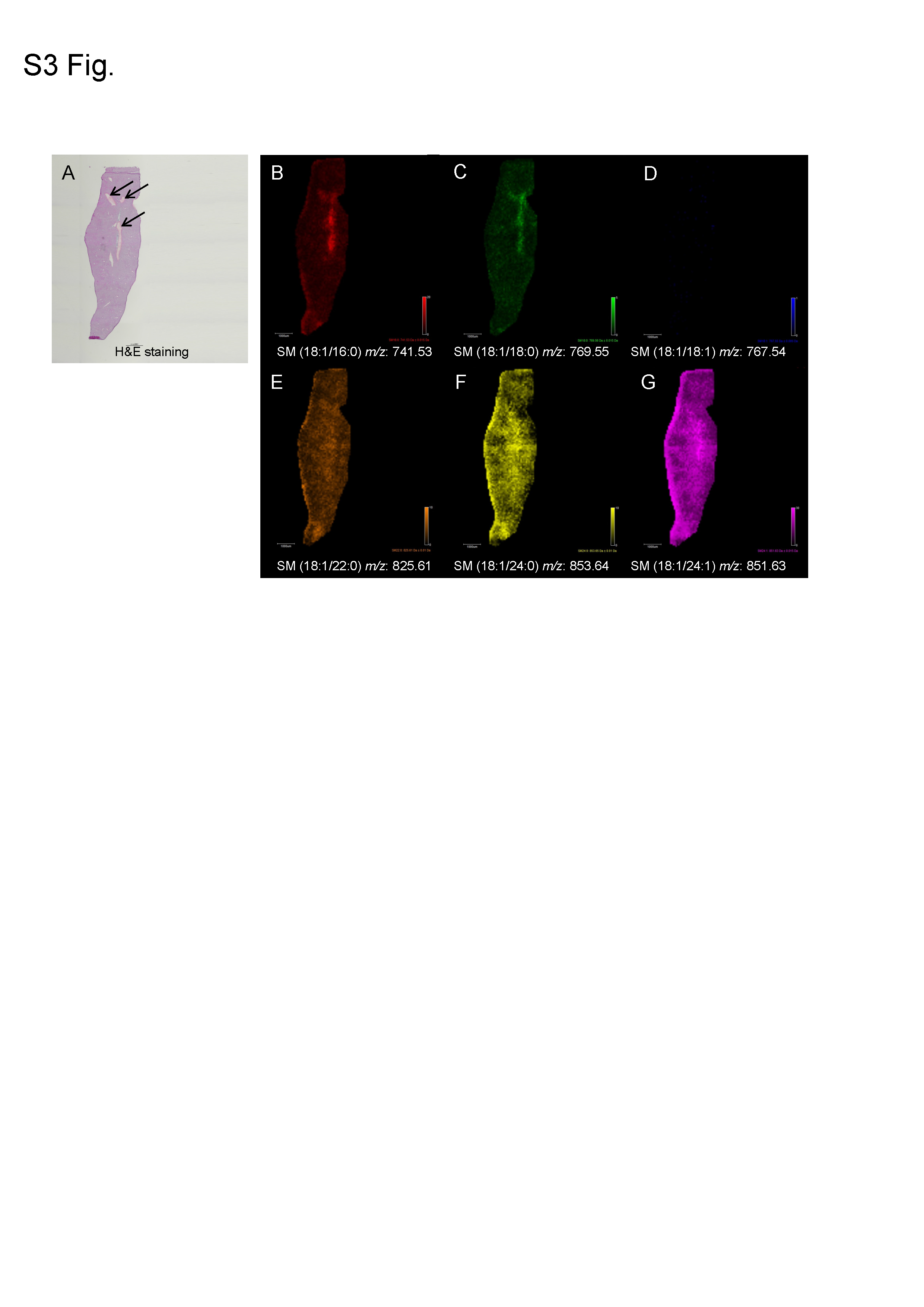

Supplement: S3 Fig — (A) Representative image of a liver section with H&E staining. Arrows indicate the region of the vessels. Representative images of (B) [SM (d18:1/16:0) +K]+, (C) [SM (d18:1/18:0) +K]+, (D) [SM (d18:1/18:1) +K]+, (E) [SM (d18:1/22:0) +K]+, (F) [SM (d18:1/24:0) +K]+, and (G) [SM (d18:1/24:1) +K]+ are shown. Scale bar = 1 mm. Data were obtained from two individuals. (TIFF) [file pone.0152191.s003.tiff]

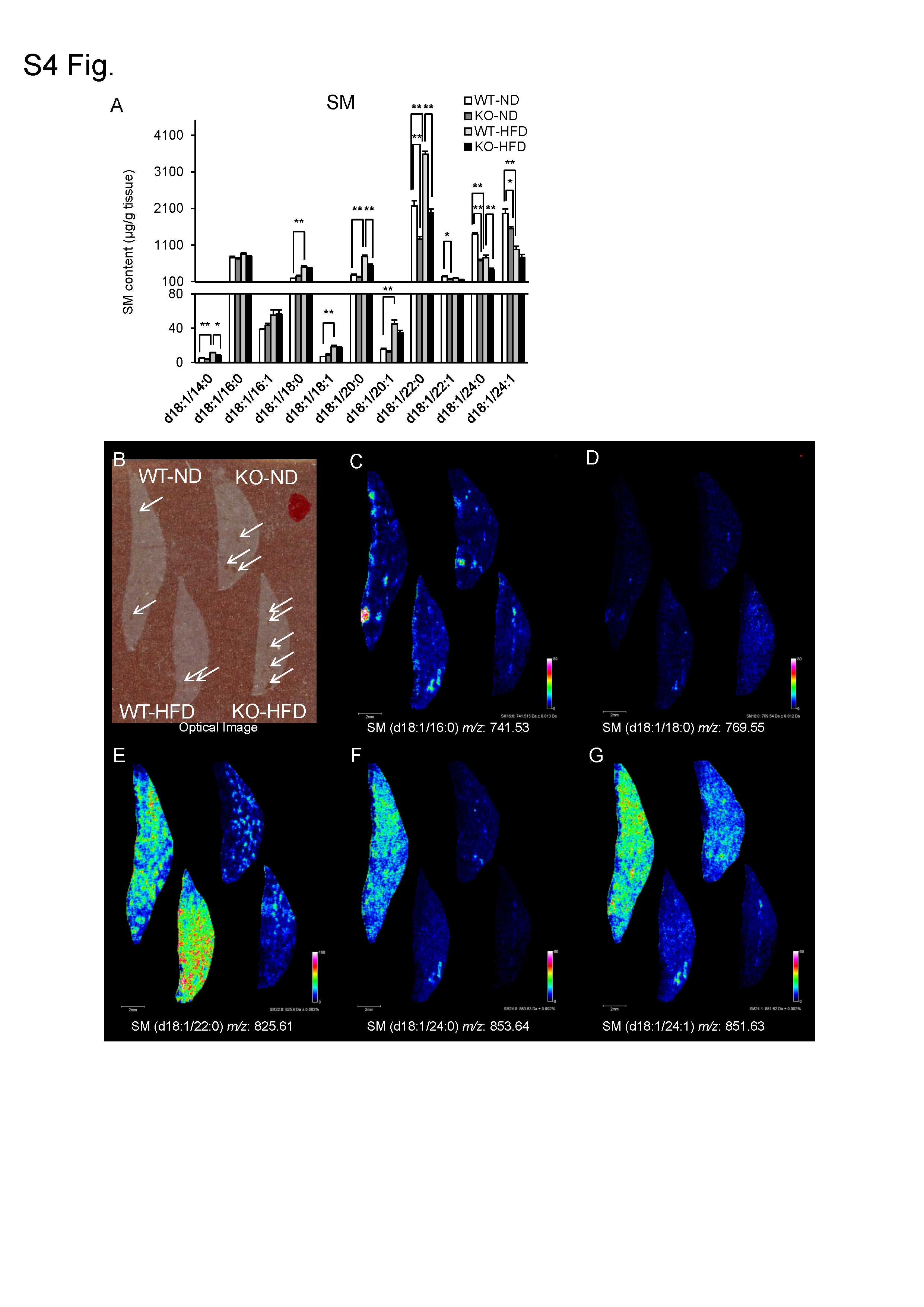

Supplement: S4 Fig — (A) Measurement by LC/ESI-MS/MS of SMs in the liver extracts of mice fed with a ND or a HFD. The levels of SMs were normalized with internal standards, SM (d18:1/16:0-d31), and expressed as μg per g tissue. Data are means ± SEM; n = 3 per group. Significant differences compared with a corresponding value in WT mice are shown. *p < 0.05, ** p < 0.01, two-way ANOVA followed by post hoc Tukey-Kramer test. (B) Representative optical images of liver sections obtained from each group of mice. Arrows indicate the region of the vessels. Representative images of (C) [SM (d18:1/16:0) +K]+, (D) [SM (d18:1/18:0) +K]+, (E) [SM (d18:1/22:0) +K]+, (F) [SM (d18:1/24:0) +K]+, and (G) [SM (d18:1/24:1) +K]+ are shown. Scale bar = 2 mm. Data were obtained from three individuals per group. (TIFF) [file pone.0152191.s004.tiff]

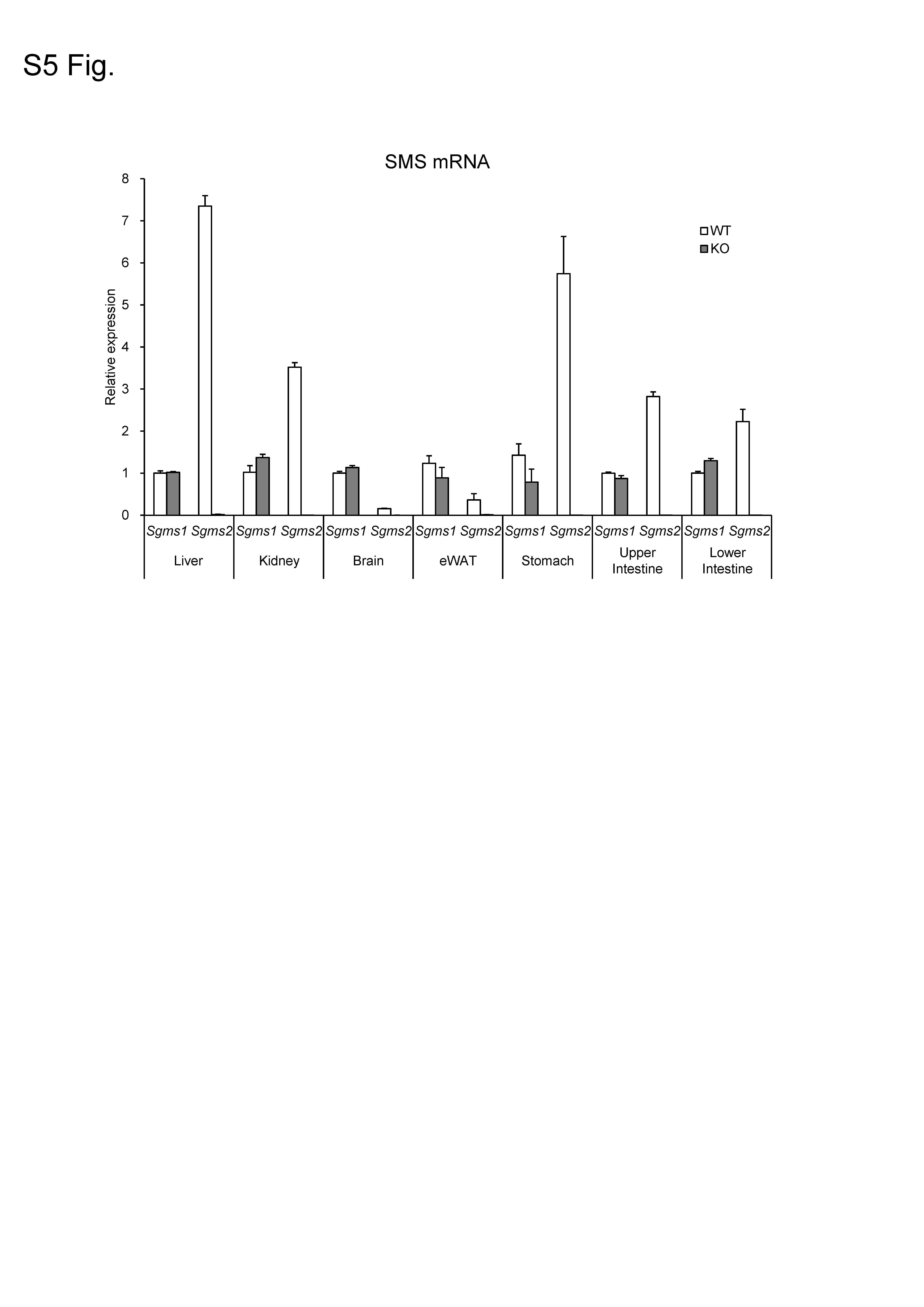

Supplement: S5 Fig — Expression of Gapdh was used as the endogenous reference for each sample. Each gene was expressed as a value relative to its expression of Sgms1 in WT mice. Data are mean ± SEM; n = 3 per group. (TIFF) [file pone.0152191.s005.tiff]

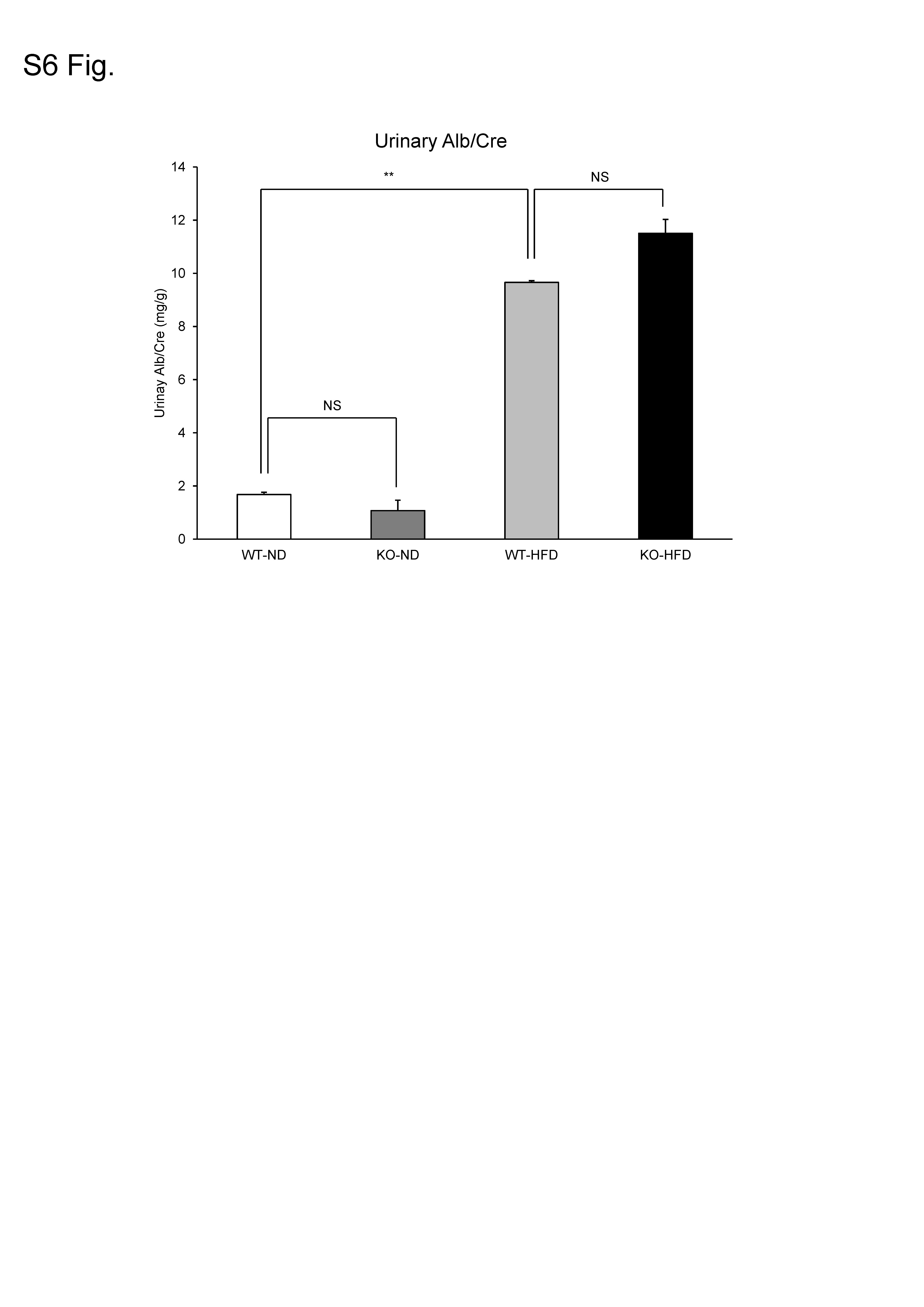

Supplement: S6 Fig — Data are mean ± SEM; n = 8–12 per group. Significant differences compared with a corresponding value in WT mice are shown. *p < 0.05, **p < 0.01, two-way ANOVA followed by post hoc Tukey-Kramer test. (TIFF) [file pone.0152191.s006.tiff]
