# Supplementary material for: Imaging Mass Spectrometry Reveals Acyl-Chain- and Region-Specific Sphingolipid Metabolism in the Kidneys of Sphingomyelin Synthase 2-Deficient Mice
Source: PLoS One. 2016 Mar 24;11(3):e0152191. doi: 10.1371/journal.pone.0152191 (PMC4806983; doi:10.1371/journal.pone.0152191)
Supplement: S1 Table — (DOCX) [file pone.0152191.s007.docx]

**S1 Table. Summary of primer sets for quantitative real-time PCR.**

| **Primers** | **Sequences 5′–3′** |
| --- | --- |
| *18S* Forward | TTGTGGCCAACGGTCTAGACAAC |
| *18S* Reverse | CCAGTGGTCTTGGTGTGCTGA |
| *Gapdh* Forward | TGTGTCCGTCGTGGATCTGA |
| *Gapdh* Reverse | TTGCTGTTGAAGTCGCAGGAG |
| *CerS1* Forward | GGTCAGATGCGTGAACTGGAA |
| *CerS1* Reverse | GGATAGAGTCCTGGATGGCTGAA |
| *CerS2* Forward | CGTGTCTATGCCAAAGCCTCA |
| *CerS2* Reverse | GTCTGGTAGAAATGTTCCAAGGTG |
| *CerS3* Forward | GCTGGATGGAAGCAGACGTGTA |
| *CerS3* Reverse | TAGTGCAAAGGCAGGATCAGAGTG |
| *CerS4* Forward | CATGACTGCTCCGACTACCTG |
| *CerS4* Reverse | GAATATGAGGCGCGTGTAGAA |
| *CerS5* Forward | ACGTGAGCGGCTCTGTACCA |
| *CerS5* Reverse | GAGCACCTGCAGGATCAGGA |
| *CerS6* Forward | CACCTGGGCAGACCTGAAGA |
| *CerS6* Reverse | TGGCACATGGTTTGGCTATGA |
| *Sgms1* Forward | ACCACCGTGTGCTTCTGTATCCTA |
| *Sgms1* Reverse | AAATGGCTTACAGCCCTGTCTTTG |
| *Sgms2* Forward | GATTACTTCGACCGGGTCAA |
| *Sgms2* Reverse | GCACAGGTAACGTAGTGACA |
| *Elovl1* Forward | TCCCTTTGAACCCTTCACTGCT |
| *Elovl1* Reverse | ATCAGCTCGTGGTACAAGTTCACAA |
| *Elovl2* Forward | GCAGCTGAAGGCCTTTGATAATG |
| *Elovl2* Reverse | AGGTAAGAGTCCAGCAGGAACCAC |
| *Elovl3* Forward | TGGTCCTTCTTCCTGGCAAT |
| *Elovl3* Reverse | AGGATGATGAAGGCCGTGT |
| *Elovl4* Forward | GAGTTCTATCGCTGGACCTGGAC |
| *Elovl4* Reverse | AGAGCGTGCTTATGCTTATCGTTG |
| *Elovl5* Forward | ACATCTGGTGGTTTGTGATGAACTG |
| *Elovl5* Reverse | TGAGGACATGGATGAAGCTGTTG |
| *Elovl6* Forward | TCAACGAGAACGAAGCCATCC |
| *Elovl6* Reverse | AGTCAGCGACCAGAGCACGA |
| *Elovl7* Forward | CAATGGGACCAGCCTACCAGA |
| *Elovl7* Reverse | TCTGGCCTATGTGGATAGTGACAAG |
